# Draft Genome Sequences for Five Strains of *Trabulsiella odontotermitis*, Isolated from *Heterotermes* sp. Termite Gut

**DOI:** 10.1128/genomeA.01289-15

**Published:** 2015-11-05

**Authors:** Myrna Olvera-García, Héctor Fontes-Perez, America Chávez-Martínez, Oscar Ruiz Barrera, Felipe A. Rodríguez-Almeida, Alejandro Sanchez-Flores, Agustín Corral-Luna

**Affiliations:** a Facultad de Zootecnia y Ecología, Universidad Autónoma de Chihuahua, Chihuahua, México; b Unidad de Secuenciación Masiva y Bioinformática, Instituto de Biotecnología, Universidad Nacional Autónoma de México, Cuernavaca, Morelos, México

## Abstract

*Trabulsiella odontotermitis* represents a novel species in the genus *Trabulsiella* with no complete genome reported yet. Here, we describe the draft genome sequences of five isolates from termites present in the north of Mexico, which have an interesting pool of genes related to cellulose degradation with biotechnological application.

## GENOME ANNOUNCEMENT

Cellulose is the most abundant polysaccharide in the plant cell wall (1). This linear homopolymer of glucose linked by β-1,4 glycosidic bonds cannot be digested by most animals, but termites can use it as a main energy source, due to the cellulolytic enzymes produced and secreted by their gut microbiota (2, 3). The most important cellulases are endoglucanase (endo-β-1,4-glucanase, EC 3.2.1.4), cellobiohydrolase (exo-β-1,4-glucanase, EC 3.2.1.91), and β-glucosidase (EC 3.2.1.21), which are employed in a wide range of biotechnological processes, including those in the textile, paper, and biofuel industries (4), as well as in animal food formulation (5). We isolate and characterized five bacterial strains, with significant cellulolytic activity, from *Heterotermes* sp. termite gut found in the north of Mexico. Therefore, to characterize them at a species level and to study their cellulolytic potential, we sequenced and reconstructed their genomes.

Total DNA was extracted for each isolate using the NucleoSpin genomic DNA purification kit (Macherey Nagel, Düren, Germany). Illumina sequencing libraries were prepared following the vendor’s protocol using the Illumina GAIIx platform. The yield and estimated coverage are depicted in Table 1. The assembly was performed using ABySS version 1.3.5 (6) for each strain using a *k*-mer size of 47, 51, 25, 47, and 39, respectively. The average of GC content for each strain was ~55%, which is similar to other *Trabulsiella* spp. High-quality draft genomes were obtained using Reapr version 3, SSPACE version 3, and GapFiller version 1.10 (7–9) for misassembly error correction, scaffolding and scaffold gap filling, respectively. Gene prediction was performed using GeneMarkS with default options (10). The annotation was performed adapting the Trinotate pipeline (http://trinotate.github.io). The statistics for each *T. odontotermitis* draft genome are summarized in Table 1.

**TABLE 1:** Assembly statistics for the draft genomes

Strain	Accession number	Yield (millions of reads)	Coverage	No. of contigs	Avg contig length (kb)	*N*_50_ (kb) / *N*_90_ (kb)	Genome size (Mb)	No. of CDSs^*a*^
TbO1.1	LIFU00000000	3.5	54.5×	50	92.40	29.98/94.98	4.62	4,224
TbO2.3	LIFV00000000	4.1	69×	40	115.04	29.97/183.19	4.60	4,221
TbO2.7	LIFW00000000	1.8	28.5×	76	59.98	135.75/40.86	4.55	4,255
TbOT1.10	LIFX00000000	4.4	68.6×	45	102.63	310.00/95.22	4.62	4,235
TbOT1.3	LIFY00000000	4.2	66×	42	109.29	300.73/183.80	4.59	4,232

a CDSs, coding sequences.

Functional annotation in all strains confirmed the presence of genes related to cellulose hydrolysis, specifically endo-1,4-d-glucanase, β-glucosidase, and cellulase-β-glucosidase activities. However, it is necessary to further characterize them at an enzymatic activity level. The genomes of these strains provide an extraordinary resource for comparative genomics among several species of this genus and also functional information to study functions related to cellulose degradation with biotechnological applications.

### Nucleotide sequence accession numbers.

This whole-genome shotgun project has been deposited at GenBank under the accession number shown in Table 1.
